# Crosstalk Between the MSI Status and Tumor Microenvironment in Colorectal Cancer

**DOI:** 10.3389/fimmu.2020.02039

**Published:** 2020-08-12

**Authors:** Anqi Lin, Jian Zhang, Peng Luo

**Affiliations:** Department of Oncology, Zhujiang Hospital, Southern Medical University, Guangzhou, China

**Keywords:** Colorectal cancer, tumor microenvironment, microsatellite instability, immune checkpoint inhibitors, colon adenocarcinoma, rectum adenocarcinoma

## Abstract

Colorectal cancer (CRC) patients, especially those with deficient mismatch repair (dMMR)/microsatellite instability-high (MSI-H) tumors, whose sensitivity to immune checkpoint inhibitors (ICIs) is significantly higher than that of patients with microsatellite-stable (MSS)/microsatellite instability-low (MSI-L) tumors, have derived clinical benefits from immunotherapy. Most studies have not systematically evaluated the immune characteristics and immune microenvironments of MSI-H and MSS/MSI-L CRCs. We analyzed the relationship between the MSI status and prognosis of ICI treatment in an immunotherapy cohort. We further used mutation data for the immunotherapy and The Cancer Genome Atlas (TCGA)-CRC [colon adenocarcinoma (COAD) + rectum adenocarcinoma (READ)] cohorts. For mRNA expression, mutation data analysis of the immune microenvironment and immunogenicity under different MSI statuses was performed. Compared with CRC patients with MSS/MSI-L tumors, those with MSI-H tumors significantly benefited from ICI treatment. MSI-H CRC had more immune cell infiltration, higher expression of immune-related genes, and higher immunogenicity than MSS/MSI-L CRC. The MANTIS score, which is used to predict the MSI status, was positively correlated with immune cells, immune-related genes, and immunogenicity. In addition, subtype analysis showed that COAD and READ might have different immune microenvironments. MSI-H CRC may have an inflammatory tumor microenvironment and increased sensitivity to ICIs. Unlike those of MSI-H READ, the immune characteristics of MSI-H COAD may be consistent with those of MSI-H CRC.

## Introduction

In recent years, anti-PD-(L)1 antibodies have served as representative immune checkpoint inhibitors (ICIs) and have brought a new dawn in the treatment of advanced melanoma, non-small cell lung cancer, bladder cancer, and other solid tumors (1–3). The frequency of deficient mismatch repair (dMMR)/microsatellite instability-high (MSI-H) tumors in Colorectal cancer (CRC) is approximately 15%, and stage IV dMMR/MSI-H tumors constitute only ∼2–4% of all metastatic CRCs (mCRCs) (4, 5). CRC patients may also benefit from immunotherapy, especially CRC patients with dMMR/MSI-H tumors, who are significantly more sensitive to ICIs than CRC patients with microsatellite-stable (MSS)/microsatellite instability-low (MSI-L) tumors (6, 7). The KEYNOTE-016 study showed that 62% (7/13) of patients with MSI-H CRC pretreated with ICIs achieved an objective response and did not reach the median for progression-free survival (PFS) or overall survival (OS) (6). Moreover, no MSS/MSI-L patients achieved an objective response, but they had median PFS and OS times of only 2.2 and 5.0 months, respectively. Another study showed that patients with MSI-H CRC had a 60% objective response rate (ORR) and an 84% disease control rate (DCR) after receiving ICIs. At the cutoff time, 82% of tumor responses were ongoing, and 74% of treatment responses lasted more than 6 months; the median PFS of all 45 patients had not yet been reached, the 12-month PFS rate was 77%, and the 12-month OS rate was 83% (8). Therefore, the FDA approved dMMR/MSI-H as a biomarker for MSI-H/dMMR tumors (5).

The MSI status may change the tumor microenvironment (TME) of CRC patients from multiple aspects, thereby affecting the efficacy of ICIs in CRC patients. With a deeper understanding of the factors influencing CRC immunotherapy outcomes, we note that compared with MSS/MSI-L CRC, with a low tumor mutational burden (TMB; <8 mutations/10^6^ DNA bases), MSI-H CRC has a higher TMB (>12 mutations/10^6^ DNA bases) (5). In addition, MSI-H CRC has more immune cell infiltration [especially tumor-infiltrating lymphocytes (TILs) and type I interferons], which is associated with a better prognosis (5, 9). A Th17-type, IL-17-dominant TME indicates a poor prognosis (10). However, most studies have not systematically evaluated differences in the immune microenvironment between MSI-H and MSS/MSI-L CRCs (5).

In this article, we systematically analyzed the differences between MSI-H and MSS/MSI-L CRCs and their subtypes [colon adenocarcinoma (COAD) and rectum adenocarcinoma (READ)] in regard to the TME, immunogenicity, immune-related gene expression profiles (GEPs), and signaling pathways. Consistent with previous studies, patients with MSI-H CRC benefited more from ICIs than patients with MSS/MSI-L CRC. Combined with gene set enrichment analysis (GSEA) of the MSI status, antitumor immunity and the possible mechanism underlying the prognostic differences among CRC patients receiving ICIs in relation to the TME were elucidated to provide theoretical guidance for further improving the curative effect of ICI treatment on MSI-H CRC patients in the future and solve the problems underlying why MSS/MSI-L CRC patients do not benefit from ICIs.

## Materials and Methods

### Data Sources

To explore the factors that affect the prognosis of ICIs in patients with different MSI statuses, we used cBioPortal^1^ to download a published clinical cohort (11) of CRC patients receiving ICIs (Samstein et al.). Mutation data sequenced by the MSK-IMPACT panel and clinical data were used for further analysis. In the ICI-treated cohort, we defined MSI scores ≥10 as MSI-H and MSI scores <10 as MSS/MSI-L (12). The R package “TCGAbiolinks” (13) was used to download the clinical and sample information (mRNA expression profile, MSI status, and somatic mutation data) of The Cancer Genome Atlas (TCGA)-COAD and TCGA-READ datasets from the Genomic Data Commons^2^. The gene expression units of both the TCGA-COAD and TCGA-READ datasets were log2[FPKM] + 1 (13). Subsequently, TCGA-COAD and TCGA-READ were combined into a TCGA-CRC dataset for subsequent analysis.

In addition, we downloaded microarray data (GSE24551) from the NCBI Gene Expression Omnibus (GEO) database. The annotation of gene symbols was based on the corresponding probe in the GPL5175 platform. We used the “normalizeBetweenArrays” function in the “limma” (14) R package to normalize the microarray data.

Whole-exome sequencing (WES), gene expression, drug response, and MSI data for CRC cell lines were downloaded from the Genomics of Drug Sensitivity in Cancer (GDSC) database (15). The unit of drug response was the ln(IC50) value.

### Immune-Related Analysis

We used the CIBERSORT web portal^3^ (16) with default parameters to analyze mRNA expression data to estimate the abundances of 22 immune cell types in TCGA-CRC. Immune-related scores and the neoantigen load (NAL) for TCGA-CRC (17) and immune-related genes and their functional classifications were obtained from articles published by Thorsson et al. and Rooney et al. (17, 18). The MANTIS score, which predicts the MSI status of tumors, was published by Bonneville et al. (19). Non-synonymous mutations in the TCGA-COAD, TCGA-READ, and GDSC-CRC cohorts were used as the raw mutation count and divided by 38 Mb to quantify TMB (20). The R package “ComplexHeatmap” was used to visualize the genetic characteristics of the ICI-treated CRC, TCGA-CRC, and GDSC-CRC cohorts (21).

### GSEA and DNA Damage Repair Mutation Number Analysis

Gene expression data for the TCGA-CRC, TCGA-READ, TCGA-COAD, and GDSC-CRC cohorts were normalized with the R package “edgeR” and analyzed by GSEA; microarray data (GSE24551) were normalized with the R package “limma” and analyzed by GSEA. GSEA was performed with the “clusterProfiler” R package and the Molecular Signatures Database (MSigDB) to annotate the dataset, where Gene Ontology (GO), Kyoto Encyclopedia of Genes and Genomes (KEGG), and Reactome terms were considered significant at *P* < 0.05. The gene sets generated by GSEA and DNA Damage Repair (DDR) analysis were obtained from the MSigDB of the Broad Institute (22) (Supplementary Table S1).

### Statistical Analysis

The Mann–Whitney *U* test was used to compare differences between two independent groups when the dependent variable was not normally distributed (including TMB, NAL, DDR mutations, immune-related gene expression levels, and immune-related scores). Fisher’s exact test was used to compare the mutation status of the genes with the top 20 mutation rates, sex, sample type, and drug type in the ICI-treated CRC cohort between patients with MSI-H and those with MSS/MSI-L. Fisher’s exact test was also used to compare differences in the mutation status of the top 20 mutation rates, sex, race, ethnicity, clinical stage, and histological type in the TCGA-CRC cohort between MSI-H and MSS/MSI-L patients. Kaplan–Meier and log-rank tests were used to analyze OS under different MSI statuses (ICI-treated cohort: MSI scores ≥ 10/MSI scores < 10) and TMB levels (cutoff: median). The Spearman rank correlation was used to test associations between the MANTIS score and other immune-related variables. *P* < 0.05 was considered statistically significant, and all statistical tests were two sided. The chi-square test was applied to compare the difference in the proportion of MSI-H and MSS/MSI-L CRCs between the high and low DDR mutation groups. All statistical tests and visualization analyses were completed with R software.

## Results

### MSI-H Was Related to Prolonged OS After ICI Treatment

**FIGURE 1:**
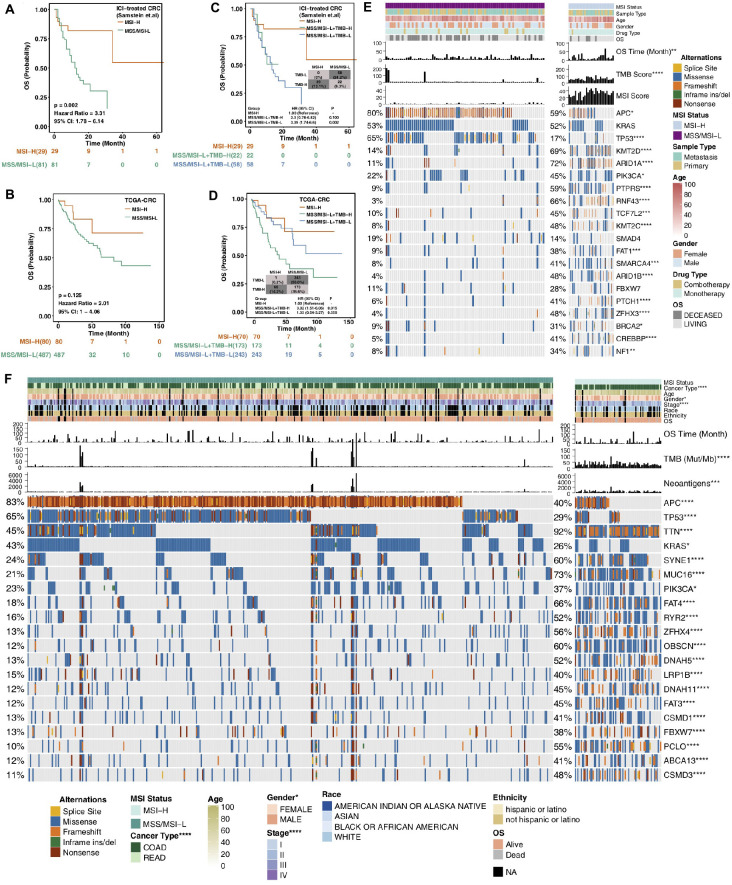
Survival curves for patients with CRC stratified by MSI status and mutational characteristics of CRC patients or cell lines stratified by MSI status. **(A,B)** Kaplan–Meier estimates of OS in the ICI-treated CRC cohort **(A)** and TCGA-CRC cohort **(B)** comparing patients with MSI-H CRC with their respective counterparts with MSS/MSI-L CRC. **(C,D)** Kaplan–Meier estimates of OS in the ICI-treated CRC cohort **(C)** and TCGA-CRC cohort **(D)** comparing patients with MSI-H CRC with their respective counterparts with MSS/MSI-L + TMB-H or MSS/MSI-L + TMB-L CRC. **(E)** Top 20 frequently mutated genes in CRC in the Samstein cohort (ICI-treated cohort). Genes are ranked by their mutation frequency in CRC patients. Mutation rates, sex, drug type, and sample type were tested by Fisher’s exact test. TMB and age were tested by the Mann–Whitney *U* test. Asterisks indicate a significant difference between MSI-H and MSS/MSI-L CRCs. **(F)** Top 20 frequently mutated genes in the TCGA-CRC cohort. Genes are ranked by their mutation frequency in CRC patients. Mutation rates, clinical stage, race, sex, cancer type, and ethnicity were tested by Fisher’s exact test. TMB, NAL and age were tested by the Mann–Whitney *U* test. CRC, colorectal cancer; TCGA, The Cancer Genome Atlas; OS, overall survival; TMB, tumor mutational burden; MSS/MSI-L, microsatellite-stable/microsatellite instability-low; MSI-H, microsatellite instability-high; and ICI, immune checkpoint inhibitor; (*P < 0.05; **P < 0.01; ***P < 0.001; and ****P < 0.0001; Mann–Whitney *U* test).

Consistent with previous research, the results obtained from the ICI-treated CRC cohort from Samstein et al. (11) showed that the MSS/MSI-L group was not sensitive to ICI treatment [log-rank test *p* = 0.002; hazard ratio (95% CI): 3.31 (1.78–6.14); Figure 1A]. With OS as the focus, the TCGA-CRC cohort survival analysis showed no significant difference between the MSI-H group and the MSS/MSI-L group (Figure 1B). Most TCGA-CRC treatments are traditional treatments, such as surgery or chemoradiation. The KEYNOTE-177 trial (NCT02563002), a randomized trial, compared first-line pembrolizumab with standard of care chemotherapy in MSI-H/dMMR mCRC. Differences in PFS were observed between CRC patients treated with chemotherapy and CRC patients treated with pembrolizumab (23). We further explored the impact of TMB on the prognosis of patients with different MSI statuses. Patients with MSI-H CRC had a higher TMB than those with MSS/MSI-L CRC (Figures 1C,D). As expected, the MSI-H CRC group was associated with a better prognosis for immunotherapy than was the MSS/MSI-L tumor mutational burden-low (TMB-L) group (*P* = 0.002; Figure 1C). However, in the TCGA-CRC cohort, compared with the MSS/MSI-L + tumor mutational burden-high (TMB-H) group, the MSI-H group experienced prolonged OS (*P* = 0.015; Figure 1D). The process of our analysis is shown in Supplementary Figure S1.

### Mutational Characteristics Based on the MSI Status

MSI is one of the important reasons for the development of CRC. It refers to an alteration or deletion of DNA repeat sequences caused by mutations in MMR genes such as MSH2, MSH6, MLH1, PMS1, and PMS2, which may result in tumor formation (24, 25). Due to the accumulation of microsatellite sequence mutations and frame shift mutations during protein translation, tumor cells produce a large number of abnormal polypeptide fragments that are relatively easily recognized by the immune system and stimulate an antitumor immune response (26). Based on the MSI status, we compared the clinical characteristics of patients to assess differences between the MSI-H and MSS/MSI-L groups. In the immunotherapy cohort, there were no significant differences in sex, sample type, drug type, or age between the MSI-H and MSS/MSI-L groups. In the TCGA-CRC cohort, COAD (93.0% vs 70.0%, *P* < 0.0001), female sex (59% vs 44%; *P* < 0.05), and early-stage disease were more often observed in the MSI-H group than in the MSS/MSI-L group.

Figure 1E shows the mutational landscape of gene mutations in ICI-treated CRC patients, indicating that MSI-H has a higher frequency of mutations than MSS/MSI-L. Except for the APC and TP53 genes, the other top 20 genes had higher mutation frequencies in the MSS/MSI-L group; however, there was no significant difference in KRAS. The types of mutations were mainly missense and frameshift mutations. Similarly, the gene mutational landscape of TCGA-CRC also showed that the genome of MSI-H was more unstable than that of MSS/MSI-L (Figure 1F). The mutation frequencies of the APC, TP53, and KRAS genes were higher in the MSS/MSI-L group; in contrast, the other genes had higher mutation frequencies in the MSI-H group. Regardless of the MSI status, the gene mutation class was mainly missense mutations. Similarly, the gene mutation landscape of the GDSC-CRC cell line also suggested that except for APC, TP53, and KRAS, the remaining genes with the top 20 mutation frequencies were more likely to be mutated in the MSI-H group than in the MSS/MSI-L group (Supplementary Figure S2A).

### Association of MSI-H With Enhanced Tumor Immunogenicity and Increased Numbers of Genetic Alterations in the DDR

**FIGURE 2:**
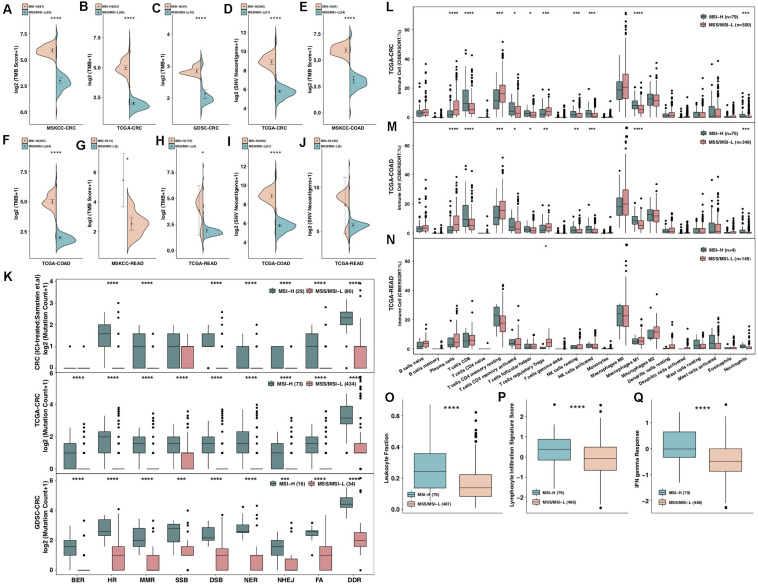
MSI-H CRC was associated with enhanced tumor immunogenicity, enriched immune cells and enhanced immune scores. **(A–C)** Comparison of TMB between MSI-H and MSS/MSI-L tumors in the ICI-treated CRC **(A)**, TCGA-CRC **(B)**, and GDSC-CRC **(C)** cohorts. **(D)** Comparison of the NAL between MSI-H and MSS/MSI-L tumors in the TCGA-CRC cohort. **(E,F)** Comparison of TMB between MSI-H and MSS/MSI-L tumors in the ICI-treated COAD **(E)** and TCGA-COAD **(F)** cohorts. **(G,H)** Comparison of TMB between MSI-H and MSS/MSI-L tumors in the ICI-treated READ **(G)** and TCGA-READ **(H)** cohorts. **(I,J)** Comparison of the NAL between MSI-H and MSS/MSI-L tumors in the TCGA-COAD **(I)** and TCGA-READ **(J)** cohorts. **(K)** Comparison of DNA damage-related gene set alterations between MSI-H and MSS/MSI-L tumors in the ICI-treated CRC, TCGA-CRC, and GDSC-CRC cohorts. **(L–N)** Comparisons of immune cells between MSI-H and MSS/MSI-L tumors in the TCGA-CRC **(L)**, TCGA-COAD **(M)**, and TCGA-READ **(N)** cohorts. **(O–Q)** Comparisons of the leukocyte fraction **(O)**, lymphocyte infiltration signature score **(P)**, and IFN-gamma response **(Q)** between MSI-H and MSS/MSI-L tumors in the TCGA-CRC cohort. GEPs were prepared using standard annotation files, and data were uploaded to the CIBERSORT web portal (http://cibersort.stanford.edu/), with the algorithm run using the LM22 signature and 1,000 permutations. FA, Fanconi anemia; HR, homologous recombination; NHEJ, non-homologous end joining; BER, base excision repair; MMR, mismatch repair; NER, nucleotide excision repair; DSB, double strand break; SSB, single strand break; TMB, tumor mutational burden; CRC: colorectal cancer; TCGA: The Cancer Genome Atlas; TMB: tumor mutational burden; MSS/MSI-L: microsatellite-stable/microsatellite instability-low; MSI-H: microsatellite instability-high; ICI: immune checkpoint inhibitor; GDSC: The Genomics of Drug Sensitivity in Cancer Project; NAL: neoantigen load; COAD: colon adenocarcinoma; and READ: rectum adenocarcinoma (*P < 0.05; **P < 0.01; ***P < 0.001; and ****P < 0.0001; Mann–Whitney *U* test).

Increased immunogenicity can cause the recruitment of dendritic cells (DCs), T cells and other immune cells to further activate the immune response, thereby exerting antitumor effects; furthermore, enhanced tumor immunogenicity (such as an increased TMB and NAL) predicts that patients can obtain long-term clinical benefits from ICIs (27, 28). Therefore, we compared the differences in tumor immunogenicity between the MSI-H group and the MSS/MSI-L group. Regardless of whether the ICI-treated, TCGA, or GDSC-CRC dataset was analyzed, the MSI-H group had a higher TMB than the MSS/MSI-L group (all *P* < 0.0001; Figures 2A–C). In addition, in the TCGA-CRC cohort, the NAL in the MSI-H CRC group was significantly higher than that in the MSS/MSI-L group (*P* < 0.05; Figure 2D). Upon exploring the relationships between the MSI status and TMB or NAL in COAD and READ, the analysis r showed that the TMB of MSI-H COAD in the ICI-treated and TCGA cohorts was significantly higher than that of MSS/MSI-L COAD (all *P* < 0.0001; Figures 2E,F). Similarly, the TMB of MSI-H READ was significantly higher than that of MSS/MSI-L READ (all *P* < 0.05; Figures 2G,H). Subgroup analysis of the NAL showed that the NAL of MSI-H COAD was significantly higher than that of MSS/MSI-L COAD (Figure 2I); however, there was no significant difference in READ (Figure 2J). The DDR system is essential for maintaining genomic integrity, and gene mutations in the DDR will result in mutations/deletions in DNA that cannot be effectively corrected and the accumulation of incorrect DNA sequences. The number of genetic mutations involved in several important pathways in the DDR system was significantly higher in the MSI-H group than in the MSS/MSI-L group for both CRC patients and CRC cell lines (all *P* < 0.0001; Figure 2K). Subgroup analysis revealed that for both COAD and READ, MSI-H patients had more mutations in genes involved in the DDR pathway than did MSS/MSI-L patients (Supplementary Figure S2B). As expected, patients with MSI-H tumors had more DDR mutations than patients with MSI-L tumors in the ICI-treated CRC, TCGA-CRC, and TCGA-COAD cohorts (all chi-square test *P* < 0.05) but not in the TCGA-READ cohort (Supplementary Figure S3).

### Association of MSI-H With an Inflamed TME

The immune microenvironment, including components such as CD8 + TILs, CD4 + TILs, Th1-type cells, and Tregs, has become one of the most important factors affecting clinical benefits in patients receiving ICIs. We used the CIBERSORT algorithm to evaluate differences in immune cells between MSI-H and MSS/MSI-L CRCs. The results showed that both MSI-H CRC and COAD had an inflammatory TME, as indicated by significantly increased numbers of plasma cells, CD8 + T cells, activated memory CD4 + T cells, follicular T helper cells, NK cells, M1 macrophages and neutrophils and significantly decreased numbers of Tregs (Figures 2L,M, all *P* < 0.05). In contrast, except for Tregs, which exhibited a significantly upregulated frequency in MSS/MSI-L READ, there were no significant differences in the remaining immune cell types between MSI-H and MSS/MSI-L CRCs (Figure 2N). Furthermore, immune-related scores were used to compare the immune status between the MSI-H and MSS/MSI-L groups (Figures 2O–Q), with the results showing that the MSI-H group had a higher leukocyte fraction score [0.24 (0.14–0.36) vs 0.14 (0.083–0.22); *P* < 0.0001], leukocyte infiltration signature score [0.38 (−0.15–0.88) vs −0.081 (−0.66–0.49); *P* < 0.0001] and IFN-gamma response [−0.0086 (−0.33–0.65) vs −0.48 (−0.87–0.0021); *P* < 0.0001].

### MSI Status and Immune GEPs

**FIGURE 3:**
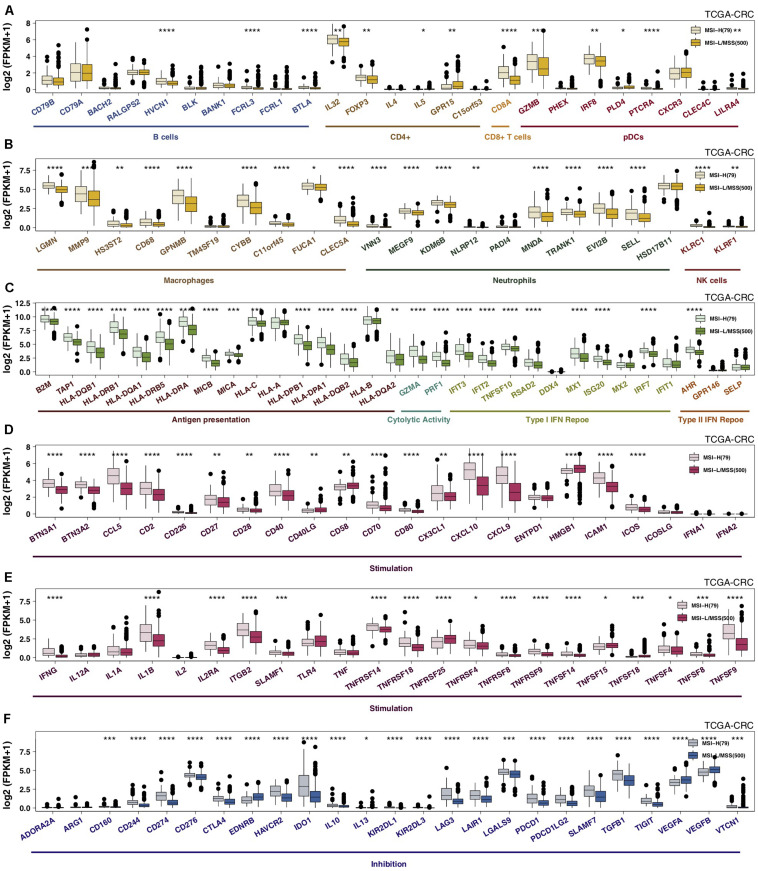
MSI-H CRC was associated with activated antitumor immunity. The expression levels of immune-related genes, such as those indicative of immune cells **(A,B)**, antigen presentation, cytolytic activity, the IFN response **(C)**, stimulation **(D,E)**, and inhibition **(F)** in MSI-H tumors vs MSS/MSI-L tumors in the TCGA-CRC cohort (*P < 0.05; **P < 0.01; ***P < 0.001; and ****P < 0.0001; **(A–F)**: Mann–Whitney *U* test]. CRC, colorectal cancer; TCGA, The Cancer Genome Atlas; TMB, tumor mutational burden; MSS/MSI-L, microsatellite-stable/microsatellite instability-low; MSI-H, microsatellite instability-high; and IFN: interferon.

Specific GEPs have become one of the most important factors influencing clinical benefits in patients receiving ICIs. Immune gene sets were used to compare GEPs between the MSI-H and MSS/MSI-L groups. We observed that the expression levels of genes related to MSI-H CRC-activated immune cells (such as B cells, CD4 + T cells, CD8 + T cells, macrophages, neutrophils, and NK cells) were significantly increased (Figures 3A,B). MSI-H CRC exhibited higher expression of genes involved in antigen presentation and cytolytic activity (CYT; CD8A, PRF1, GZMA, and GZMB) and the IFN response (Figure 3C). The results of an analysis of stimulatory immune-related genes (Figures 3D,E), such as chemokines (CX3CL1, CXCL9, and CXCL10), cytokines (IFNG, IL1B, etc.), and tumor necrosis factor receptor superfamily (TNFRSF)-related genes, indicated significant upregulation in MSI-H CRC (all *P* < 0.05). The expression of immune checkpoint genes, such as LAG3, CTLA4, CD274, PDCD1, TIGIT, IDO1, and PDCD1LG2, in MSI-H CRC was significantly higher than that in MSS/MSI-L CRC (Figure 3F; all *P* ≤ 0.05), while MSI-H CRC exhibited lower expression of VEGF. Subgroup analysis showed that regarding immune-related GEPs, MSI-H COAD was very similar to MSI-H CRC; however, MSI-READ and MSI-H CRC were completely different, and there was no significant difference in the expression of immune-related genes between MSI-H READ and MSS/MSI-L READ (Supplementary Figure S4).

### The MANTIS Score Was Linked to Improved Immune Characteristics

**FIGURE 4:**
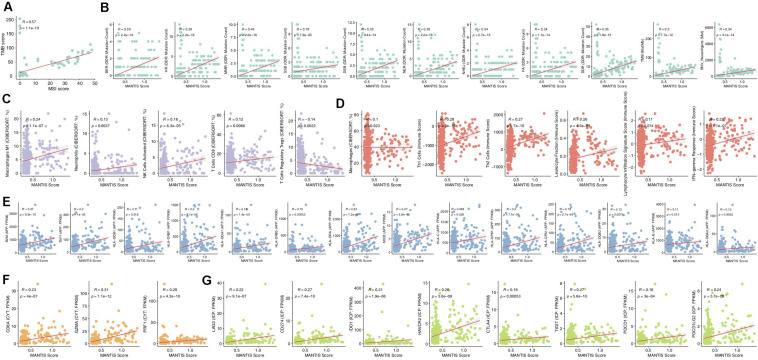
Correlations of the MANTIS score and immune-related characteristics. Correlation of the MANTIS score and TMB in the ICI-treated CRC cohort **(A)**. Correlations of the MANTIS score and tumor immunogenicity **(B)**, immune cells **(C)**, the immune score **(D)**, an APP-related gene **(E)**, a CYT-related gene **(F)**, and an ICP-related **(G)** gene in the TCGA-CRC cohort. CRC, colorectal cancer; TCGA, The Cancer Genome Atlas; TMB, tumor mutational burden; MSS/MSI-L, microsatellite-stable/microsatellite instability-low; MSI-H, microsatellite instability-high; IFN, interferon; CYT, cytolytic activity; APP, antigen processing and presentation; and ICP, immune checkpoint.

The MANTIS score is a score that predicts a patient’s MSI status and was presented in an article published by Bonneville et al. (19). The higher the MANTIS score is, the more likely a patient is to have the MSI-H status. In the ICI-treated CRC cohort, the MANTIS score was positively correlated with TMB (*P* < 0.001; Figure 4A). Similarly, in the TCGA-CRC dataset, the MANTIS score was positively related to increased immunogenicity (such as an increased TMB, NAL, or number of mutations in the DDR pathway; Figure 4B), the abundances of immune cells (such as M1 macrophages, neutrophils, activated NK cells, CD8 + T cells, and macrophages; Figure 4C), immune correlation scores (Th1 cells, Th2 cells, leukocyte fraction, leukocyte infiltration signature score, and IFN-gamma response; Figure 4D), the expression of antigen presentation-related genes (Figure 4E), the expression of CYT-related genes (Figure 4F), and the expression of immune checkpoint genes. In contrast, the MANTIS score was negatively correlated with Tregs (*R* = −0.14; *P* = 0.0022; Figure 4C).

### Comparison of Transcriptomic Traits Between MSI-H and MSS/MSI-L CRCs

**FIGURE 5:**
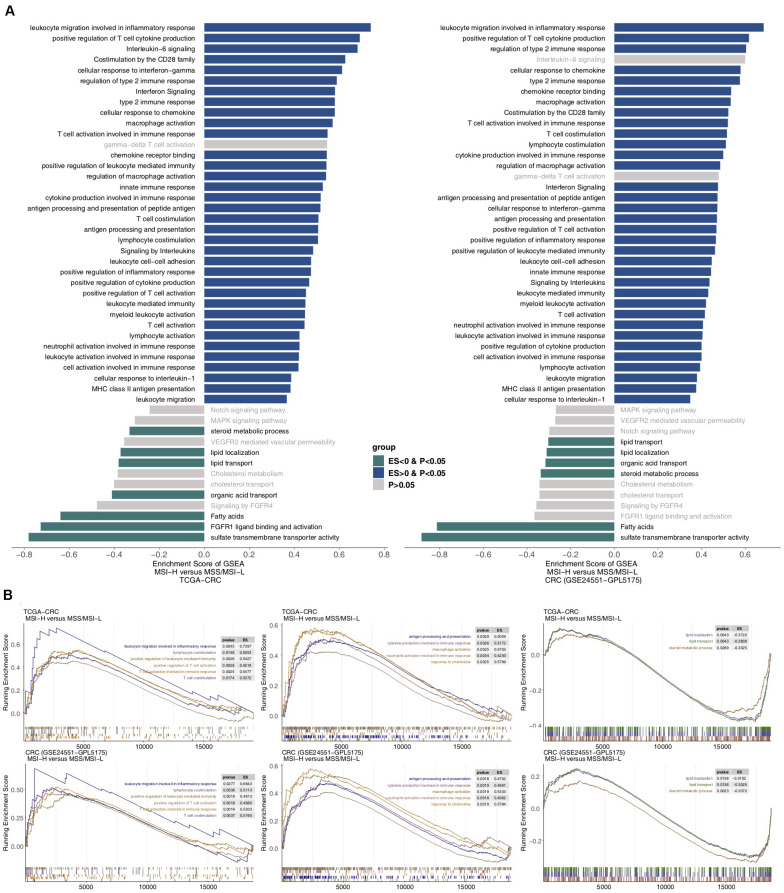
Transcriptomic analysis of the biological function traits of MSI-H and MSS/MSI-L tumors in the TCGA-CRC cohort and another CRC cohort (GSE24551). **(A)** Differences in pathway activities scored by GSEA between MSI-H and MSS/MSI-L tumors in the TCGA-CRC cohort. Enrichment results with significant differences between MSI-H and MSS/MSI-L tumors are shown. A blue bar indicates that the ES of the pathway is more than 0, while a green bar indicates that the ES of the pathway is less than 0. **(B)** GSEA of hallmark gene sets downloaded from the MSigDB. All transcripts are ranked by the log2 (fold change) between MSI-H and MSS/MSI-L tumors in the TCGA-CRC cohort and another CRC cohort (GSE24551). Each run was performed with 1,000 permutations. Enrichment results with significant differences between MSI-H and MSS/MSI-L tumors are shown. GSEA, gene set enrichment analysis; CRC, colorectal cancer; TCGA, The Cancer Genome Atlas; TMB, tumor mutational burden; MSS/MSI-L, microsatellite-stable/microsatellite instability-low; MSI-H, microsatellite instability-high; ES, enrichment score; and MSigDB, Molecular Signatures Database.

To further analyze the differences in potential biological mechanisms between MSI-H and MSS/MSI-L tumors (Figure 5), we performed GSEA on the TCGA-CRC and GEO-CRC cohorts (GSE24551-GPL5175) and intersected the enriched pathways. Figure 5A shows that the immune response-related pathways in the TCGA and GEO datasets, such as leukocyte migration involved in the inflammatory response, cellular response to IFN-gamma, and T cell activation involved in the immune response, were significantly enriched in MSI-H CRC. Pathways and metabolism-related pathways were significantly downregulated in MSS/MSI-L CRC. Figure 5B shows that immune response pathways involved in lymphocytes and T cells were significantly enriched in MSI-H CRC in the TCGA and GEO datasets [all enrichment scores (ES) > 0, *P* < 0.05]. In addition, immune response pathways involved in antigen presentation, cytokine- or chemokine-related processes and macrophage or neutrophil activity were significantly enriched in MSI-H CRC in the TCGA and GEO datasets (all ES > 0, *P* < 0.05). In contrast, lipid localization, lipid transport, and steroid metabolism processes were significantly downregulated in MSI-H CRC in the TCGA and GEO datasets (all ES < 0, *P* < 0.05). Subsequently, we analyzed COAD using GSEA or different MSI statuses in READ. The enrichment in functional signaling pathways under normal conditions showed that similar to MSI-H CRC, MSI-H COAD also showed significant upregulation of immune-related pathways and significant downregulation of metabolic pathways. In contrast, MSI-H READ behaved differently from MSI-H COAD or CRC (Supplementary Figure S5).

## Discussion

**FIGURE 6:**
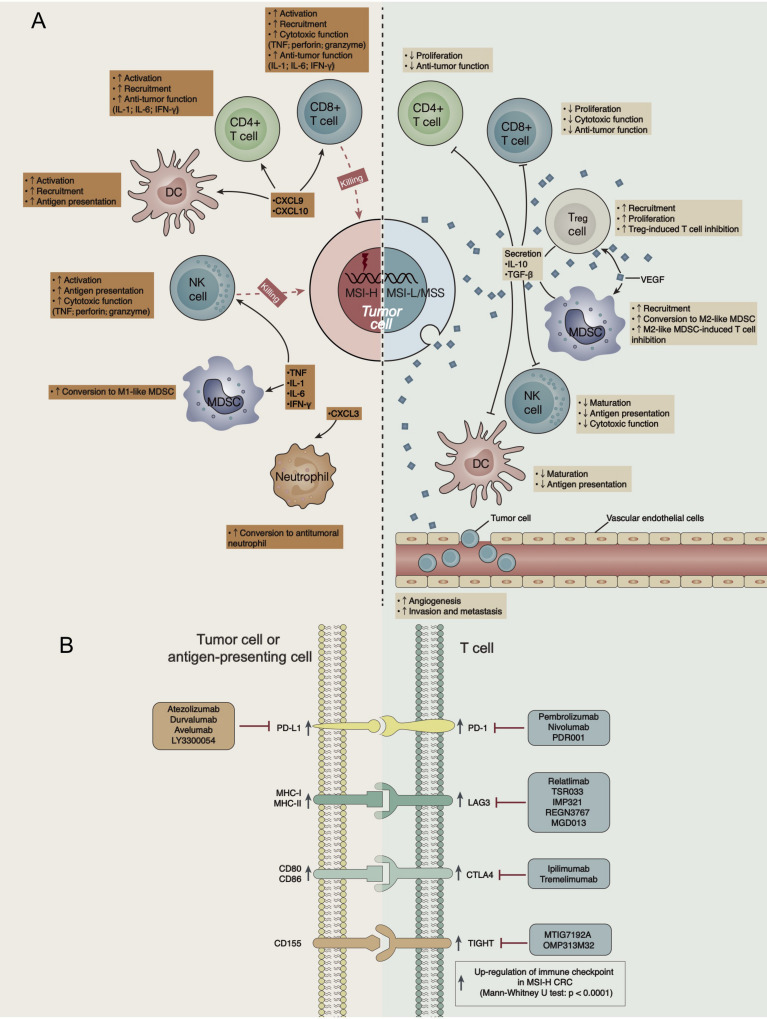
**(A)** Possible mechanism underlying the difference in the efficacy of ICI treatment in CRC patients with different MSI statuses. **(B)** The high expression of immune checkpoints in the GEP of MSI-H CRC indicates targets for ICIs.

Colorectal cancer is a common tumor of the digestive system. Although OS has been improved in recent years through combinations of treatments such as surgery, radiotherapy, chemotherapy, and targeted therapy, the overall therapeutic efficacy is still poor, and the 5-year survival rate of patients with advanced mCRC is approximately 12.5% (29). In recent years, ICIs [such as anti-PD-(L)1 antibodies] have demonstrated significant clinical effects on patients with MSI-H CRC but little effect on patients with MSS/MSI-L CRC. At present, the mechanism underlying the difference in the curative effect of ICIs between MSI-H and MSS/MSI-L CRCs is unclear. Therefore, we analyzed differences in the TME, immunogenicity, immune-related GEPs, and signaling pathways between MSI-H and MSS/MSI-L CRCs (CRC, COAD, and READ). ICI-treated MSI-H CRC was associated with a better prognosis than ICI-treated MSS/MSI-L CRC. We further explored possible factors affecting the prognostic difference in the effects of ICIs on different MSI statuses. We found that the prolonged OS of MSI-H patients after ICI treatment might be related to increased tumor immunogenicity (such as increased NAL, TMB, number of DDR pathway mutations and the expression of antigen processing and presentation-related genes), the significantly upregulated expression of immune-related genes (immune cell-, CYT-, cytokine-, chemokine-, and immune checkpoint-related genes), and elevated immune-related scores (leukocyte fraction score, leukocyte infiltration signature score, and IFN-gamma response). In addition, GSEA results for different MSI statuses showed that immune response-related pathways were significantly upregulated in MSI-H CRC or COAD, while metabolism-related pathways were significantly downregulated. Therefore, we summarized the possible mechanisms underlying the improved efficacy and prognosis in MSI-H patients receiving ICIs (Figure 6A).

One of the factors affecting ICI treatment outcomes is tumor immunogenicity (e.g., TMB, NAL, MSI status, genetic mutations in the DDR pathway and the presentation of neoantigens by HLA) (30–35). MSI is one of the most important causes of CRC. It refers to mutations in MMR genes, which result in the expansion or deletion of DNA repeat sequences (microsatellites) that then cause tumorigenesis (24, 25). Our research is consistent with previous research and shows that whether in CRC, COAD or READ, MSI-H tumors have a significantly higher TMB, NAL, and number of gene mutations in the DDR pathway than MSS/MSI-L tumors, as well as the upregulated expression of antigen presentation-related genes. The ORR of ICIs indicates a positive correlation with TMB in a variety of solid tumors (*P* < 0.001, *R* = 0.74). Similarly, the effect of the NAL on ICI treatment is also predictive (36). In addition, increased numbers of genetic mutations in the DDR pathway can lead to increased TMB, while relatively high non-synonymous mutation burdens indicate an improved ORR, prolonged PFS, and a long-lasting clinical response to immunotherapy (28). In addition, a large number of antigen processing and presentation-related genes exhibit significantly increased expression in MSI-H CRC and COAD, which play an important role in the recruitment of effector T cells and lymphocytes to neoantigen-expressing tumor cells and thus stimulates the body’s antitumor immune responses (26).

The TME has also become one of the most important factors affecting immunotherapy, and it includes TILs, antigen-presenting cells, Tregs, chemokines, cytokines, etc. In MSI-H CRC, chemokines such as CXCL9, CXCL10 and CXCL11 recruit and activate cytotoxic T lymphocytes (CTLs), DCs, and NK cells in the tumor tissue to exert an antitumor effect. For example, NK cells and CD8 + TILs secrete TNF, perforin and granzyme to exert cytotoxic effects (37), and CD4 + TILs secrete IL-1, IL-6, IFN-γ, and other cytokines, further activating other immune cells (38, 39). In addition, CXCL3 attracts neutrophils *in vivo* and inhibits tumor growth (40). Additionally, IL-1, IL-6, and TNF play important roles in macrophage polarization, converting myeloid-derived suppressor cells (MDSCs) into M1-like macrophages with antitumor functions (41). In contrast, MSS/MSI-L CRC has a VEGF-rich TME. For example, VEGF recruits MDSCs and promotes their conversion into M2-like macrophages, which inhibit T cell function-like macrophages (42). Similarly, VEGF plays important roles in Treg recruitment and proliferation, and Tregs inhibit the response and function of CTLs through a variety of direct or indirect mechanisms (42). VEGF promotes angiogenesis, invasion, and metastasis in tumor cells (42). In addition, M2-like macrophages and Tregs secrete inhibitory cytokines (such as IL-10 and TGF-β) and further suppress T cells (CD4 + T cells and CD8 + T cells) and antigen-presenting cells (such as DCs and NK cells) (5).

A specific GEP predicts some functions in the TME and is related to the therapeutic efficacy of ICIs (43). Consistent with previous results, the elevated expression of CD8A, GZMA, PRF1, CD8B, and GZMB in MSI-H CRC predicted increased CYT and an improved immunotherapy prognosis (43). In addition, the high expression of immune checkpoints in a GEP often suggests an improved immunotherapy prognosis (44, 45). The high expression of immune checkpoints in the GEP of MSI-H CRC indicates targets for ICIs (Figure 6B).

Evidence suggests that left-sided and right-sided CRCs exhibit different TME landscapes, further leading to distinct benefits of ICI treatment (46). Zhang et al. reported a higher proportion of NK cells associated with left-sided CRC than with right-sided CRC (46). NK cells are associated with the prolonged survival of CRC patients (46). Additionally, there are various biological and clinical differences that may affect mutational characteristics and immune infiltration between different CRC locations (such as right-sided and left-sided CRCs) (47). Consistent with a previous study, our findings indicate that the TME and immune characteristics of MSI-H COAD might be somewhat different from those of MSI-H READ (48). For example, Shen et al. revealed different molecular subtypes of CRC (48). Based on carefully collected and curated genomic and clinical data and immune-related algorithms, we determined that MSI-H CRC was significantly associated with enhanced tumor immunogenicity (including NAL, TMB, and DDR mutations) and an inflamed TME (including high expression levels of inflammatory immune-related genes, increased infiltration levels of immune cells, and upregulated immune-related pathways). There is a clear unmet need for exploring the mechanism of primary/secondary resistance to ICI treatment in some MSI-H CRCs in the future. Recently, single-cell RNA sequencing (scRNA-seq) was extensively developed, which allows the expression profiles of individual cell types to be obtained rapidly (49). It also plays an important role in identifying cell subtypes and illustrating molecular differences.

There are still some limitations to this study. First, we analyzed only one ICI-treated CRC cohort and the TCGA-CRC cohort. For MSI-H and MSS/MSI-L CRCs, there may be some bias in the comprehensive assessment of immune characteristics and the immune microenvironment. Second, the lack of transcriptomic, copy number variation, and protein-level data for the ICI-treated CRC cohort and the lack of relevant animal experiments in this study did not allow us to directly prove our hypothesis. Third, the number of CRC patients treated with ICIs was unfortunately very small. Fourth, we did not explore the mechanism of primary/secondary resistance to ICI treatment in some MSI-H CRCs, and more research involving large sample sizes and diverse ethnic groups is needed for subsequent analysis and verification. Additionally, scRNA-seq might help us reveal distinct cell subtypes and illustrate molecular differences in the future (49).

## Conclusion

Microsatellite instability-high CRC had a better immunotherapy prognosis than MSS/MSI-L CRC. MSI-H CRC was related to an inflammatory TME, the increased expression of immune-related genes, enhanced immunogenicity, and elevated immune-related scores. In contrast, MSS/MSI-L CRC was related to an inhibitory TME and the reduced expression of immune-related genes, immunogenicity, and immune-related scores. In addition, the TME and immune characteristics of MSI-H COAD might be somewhat different from those of MSI-H READ. Furthermore, we aimed to elucidate the possible mechanisms by which the TME of MSI-H and MSS/MSI-L affect the prognostic difference in CRC patients receiving ICI therapy to further improve the efficacy of ICI treatment in MSI-H CRC patients and provide theoretical guidance to address the problem of MSS/MSI-L patients not deriving clinical benefits from ICI treatment. In addition, the possible mechanism underlying the difference in the efficacy of ICI treatment based on different MSI statuses requires a series of prospective clinical studies and mechanistic explorations.

## Data Availability Statement

All datasets presented in this study are included in the article/Supplementary Material.

## Author Contributions

AL wrote the manuscript. PL and JZ designed the research. AL performed the research. AL, PL, and JZ wrote, reviewed, and edited the manuscript. All authors contributed to the article and approved the submitted version.

## Conflict of Interest

The authors declare that the research was conducted in the absence of any commercial or financial relationships that could be construed as a potential conflict of interest.

## Footnotes

1 https://www.cbioportal.org/

2 https://portal.gdc.cancer.gov/

3 https://cibersort.stanford.edu/

## Supplementary Material

FIGURE S1 Flow chart of this study.

FIGURE S2 **(A)** Top 20 frequently mutated genes in GDSC-CRC cell lines. Genes are ranked by their mutation frequency in CRC cell lines. Details of the CRC cell lines are annotated for each sample. **(B)** Comparison of DNA damage-related gene set alterations between MSI-H and MSS/MSI-L tumors in the TCGA-COAD and TCGA-READ cohorts.

FIGURE S3 Comparison of the proportion of MSI-H and MSS/MSI-L CRCs between the high and low DDR mutation groups.

FIGURE S4 Comparison of the expression of immune-related genes between MSI-H and MSS/MSI-L tumors in the TCGA-COAD and TCGA-READ cohorts.

FIGURE S5 Transcriptomic analysis of the biological function traits of MSI-H and MSS/MSI-L tumors in the TCGA-COAD, TCGA-READ and GDSC-CRC cohorts.

TABLE S1 List of genes included in the DNA damage response (DDR) gene set used for comparison analysis (MSigDB).
