# Supplementary figures and images for: Crosstalk Between the MSI Status and Tumor Microenvironment in Colorectal Cancer
Source: Front Immunol. 2020 Aug 12;11:2039. doi: 10.3389/fimmu.2020.02039 (PMC7435056; doi:10.3389/fimmu.2020.02039)

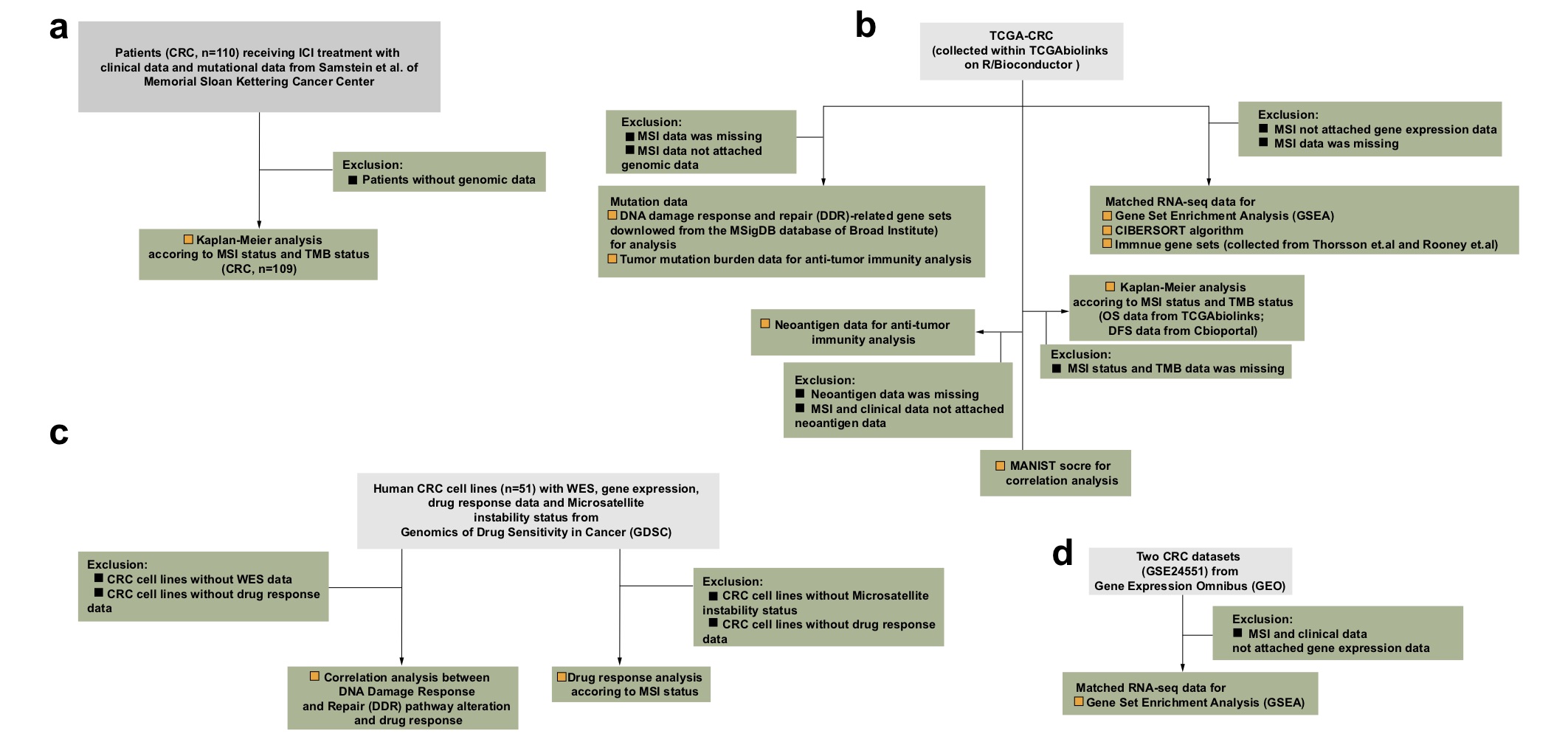

Supplement: FIGURE S1 — Flow chart of this study. [file Image_1.JPEG]

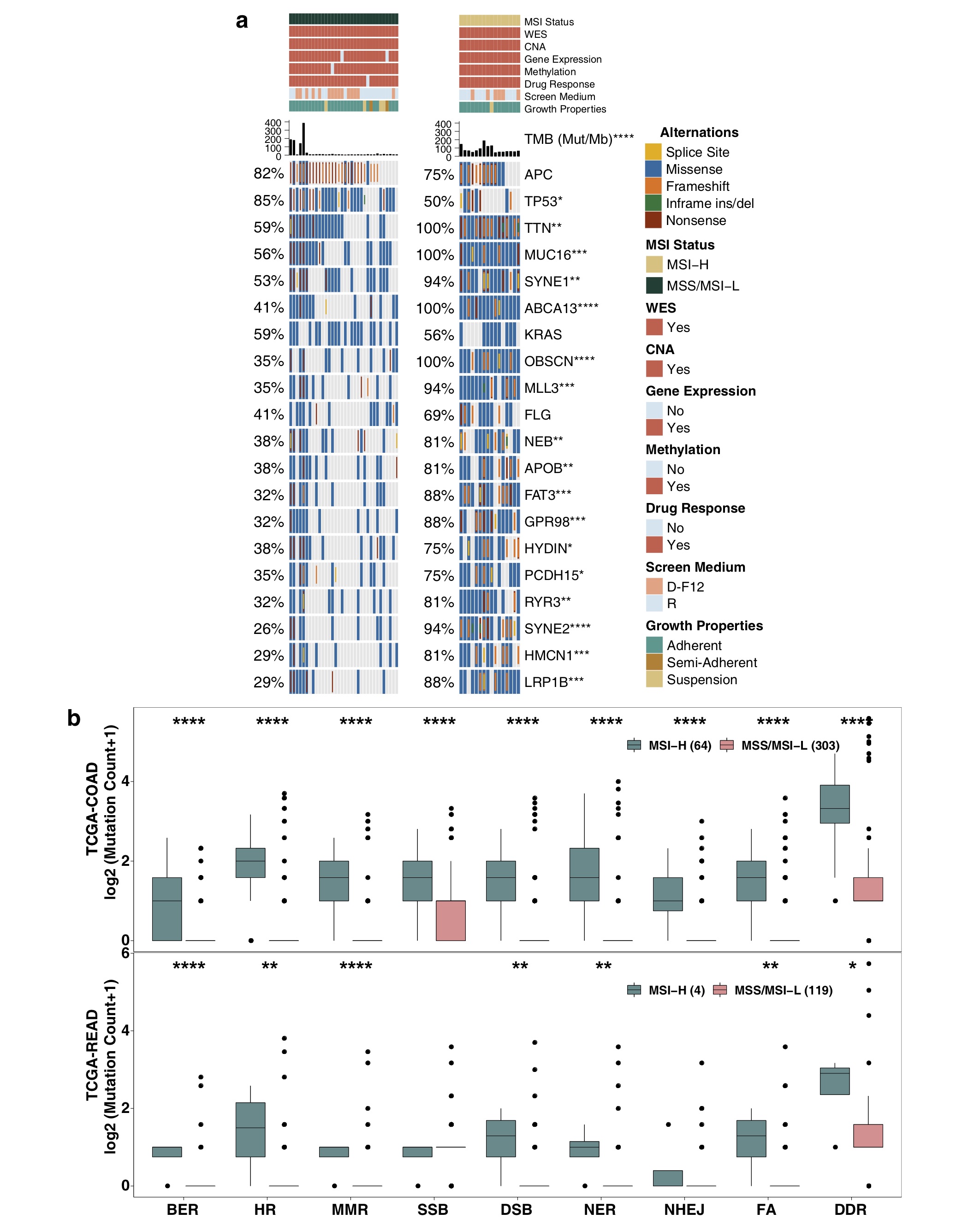

Supplement: FIGURE S2 — (A) Top 20 frequently mutated genes in GDSC-CRC cell lines. Genes are ranked by their mutation frequency in CRC cell lines. Details of the CRC cell lines are annotated for each sample. (B) Comparison of DNA damage-related gene set alterations between MSI-H and MSS/MSI-L tumors in the TCGA-COAD and TCGA-READ cohorts. [file Image_2.JPEG]

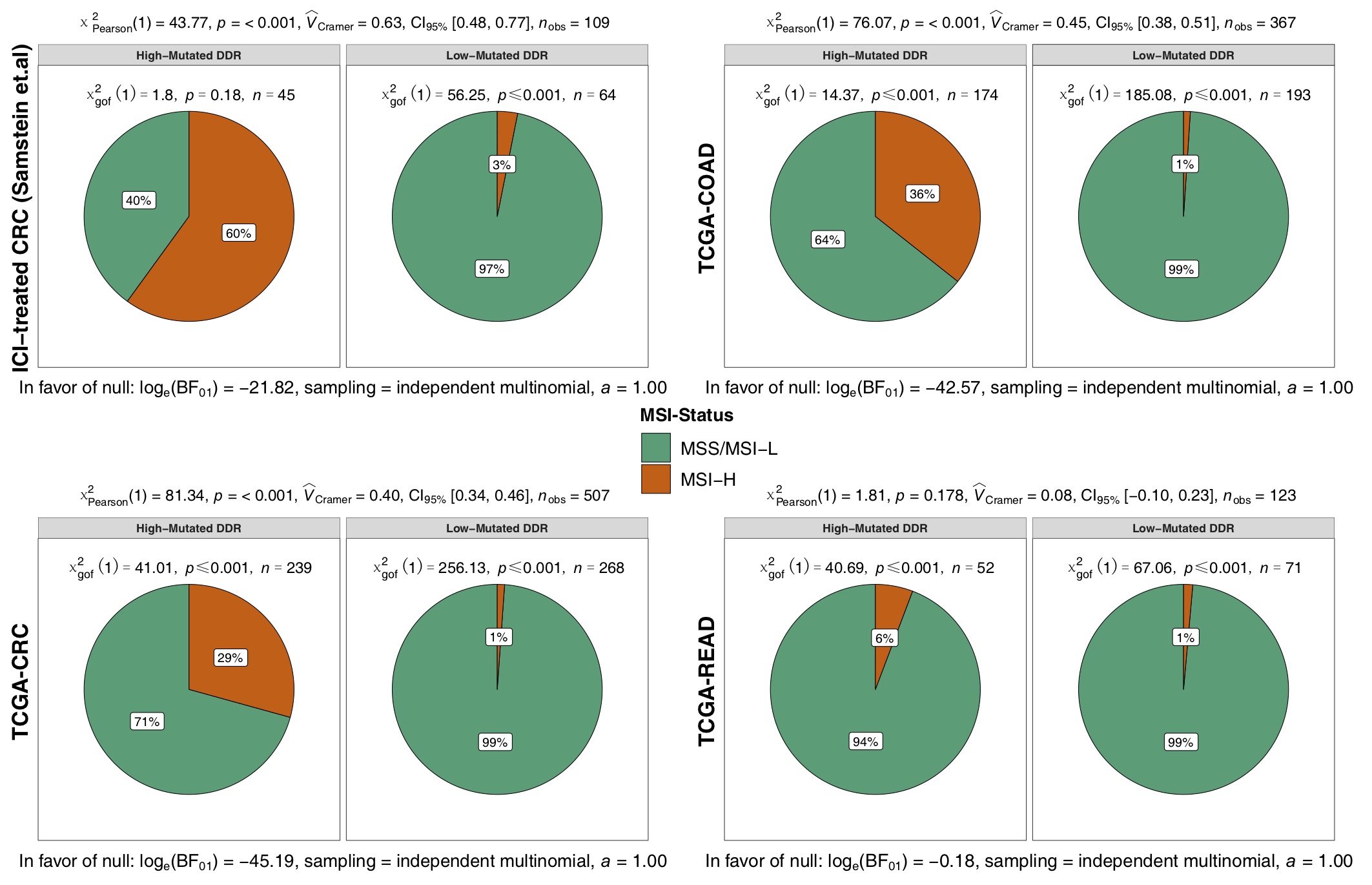

Supplement: FIGURE S3 — Comparison of the proportion of MSI-H and MSS/MSI-L CRCs between the high and low DDR mutation groups. [file Image_3.JPEG]

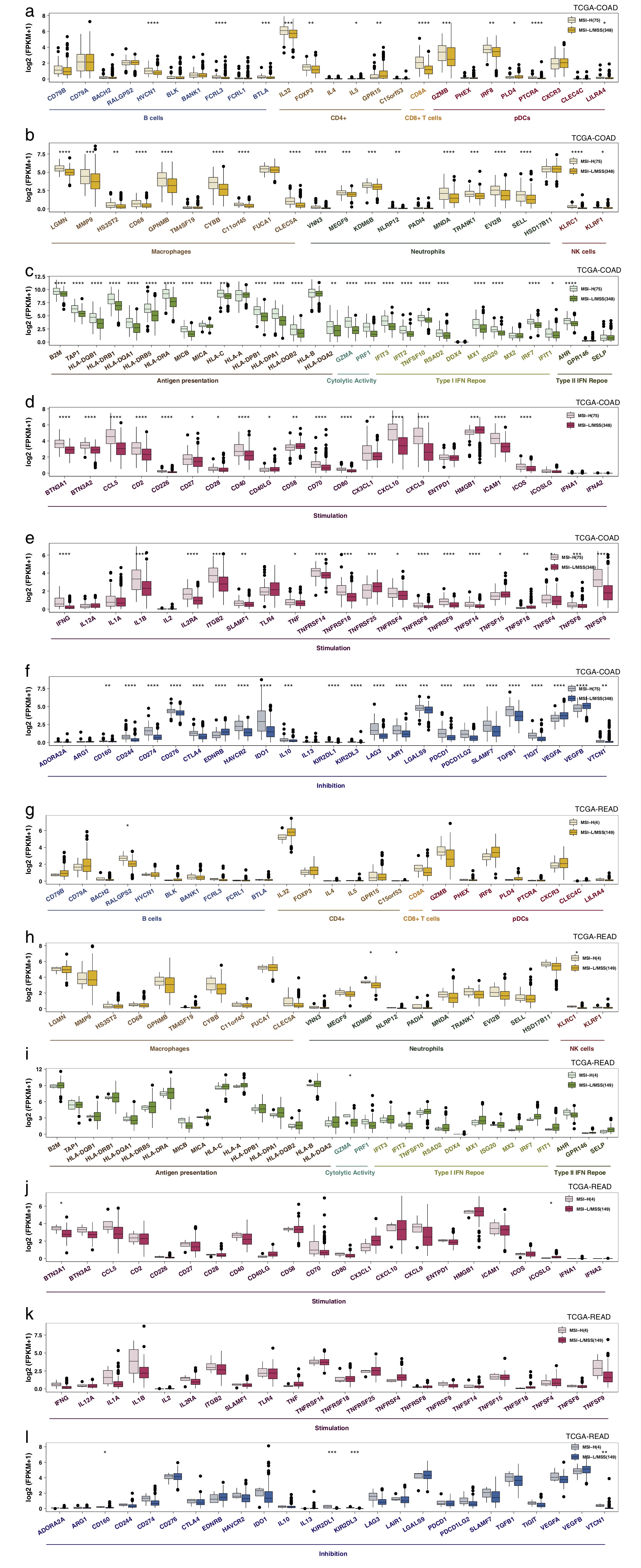

Supplement: FIGURE S4 — Comparison of the expression of immune-related genes between MSI-H and MSS/MSI-L tumors in the TCGA-COAD and TCGA-READ cohorts. [file Image_4.JPEG]

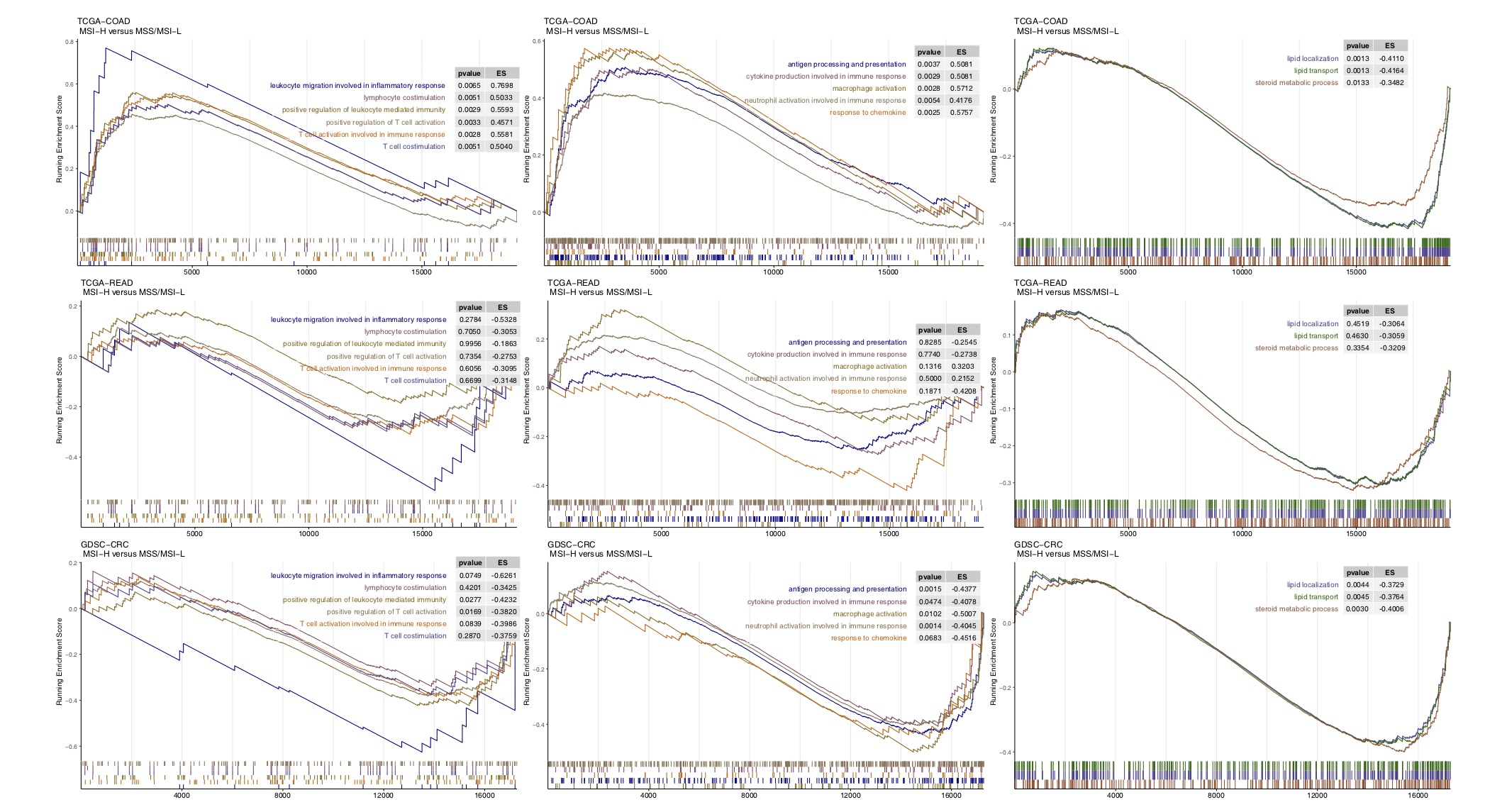

Supplement: FIGURE S5 — Transcriptomic analysis of the biological function traits of MSI-H and MSS/MSI-L tumors in the TCGA-COAD, TCGA-READ and GDSC-CRC cohorts. [file Image_5.JPEG]
