# Supplementary material for: Crosstalk Between the MSI Status and Tumor Microenvironment in Colorectal Cancer
Source: Front Immunol. 2020 Aug 12;11:2039. doi: 10.3389/fimmu.2020.02039 (PMC7435056; doi:10.3389/fimmu.2020.02039)
Supplement: TABLE S1 — List of genes included in the DNA damage response (DDR) gene set used for comparison analysis (MSigDB). [file Table_1.pdf]

S Table A1. List of genes included in DNA damage response (DDR) gene set used for Comparison Analysis (MsiDB)

Pathway	Gene Set
BER(R-HSA-73884_REACTOME_Base_Excision_Repair)	CCNO; POLD3; FEN1; SMUG1; APEX1; LIG1; LIG3; MPG; MUTYH; NTHL1; OGG1; PCNA; POLB; POLD1; POLD2; POLD4; TDG; XRCC1; MBD4
DSB(R-HSA-5696398_REACTOME_Double_Strand_Break_Repair)	RAD50; XRCC6; H2AFX; LOC389901; LIG1; LIG4; MRE11A; NBN; ATM; TDP1; PRKDC; RAD51; RAD52; RPA1; RPA2; RPA3; LOC651610; BRCA1; BRCA2; TP53BP1; XRCC4; XRCC5; BRIP1; MDC1
FA(R-HSA-6783310_REACTOME_Fanconi_Anemia_Pathway)	FANCA; FANCC; FANCD2; FANCE; FANCB; FANCF; FANCG; ZBTB32; UBE2T; ATM; ATR; FANCL; FANCM; RPS27A; LOC648152; LOC651610; LOC651921; BRCA1; BRCA2; RPS27AP11; UBA52; RPS27AP11; USP1; PALB2; C17orf70; C19orf40
HR(hsa03440_KEGG_Homologous_Recombination)	RAD50; H2AFX; LIG1; MRE11A; NBN; ATM; RAD51; RAD52; RPA1; RPA2; RPA3; LOC651610; BRCA1; BRCA2; TP53BP1; BRIP1; MDC1; RAD50; POLD3; EME1; RAD54B; RPA4; MRE11A; NBN; POLD1; POLD2; POLD4; RAD51; RAD51C; RAD51B; RAD51D; RAD52; RPA1; RPA2; RPA3; BLM; SSBP1; BRCA2; TOP3A; XRCC2; XRCC3; SHFM1; MUS81; RAD54L; TOP3B
MMR(hsa03430_KEGG_Mismatch_Repair)	POLD3; MLH3; MSH6; RPA4; LIG1; MLH1; MSH2; MSH3; PCNA; PMS2; POLD1; POLD2; POLD4; RFC1; RFC2; RFC3; RFC4; RFC5; RPA1; RPA2; RPA3; SSBP1; EXO1
NER(R-HSA-5696398_REACTOME_Nucleotide_Excision_Repair)	CDK7; POLD3; ERCC8; DDB1; DDB2; ERCC1; ERCC2; ERCC3; ERCC4; ERCC5; ERCC6; GTF2H1; GTF2H2; GTF2H3; GTF2H4; LIG1; MNAT1; PCNA; POLD1; POLD2; POLE; POLE2; POLR2A; POLR2B; POLR2C; POLR2D; POLR2E; POLR2F; POLR2G; POLR2H; POLR2I; POLR2J; POLR2K; POLR2L; XAB2; POLD4; RAD23B; RFC2; RFC3; RFC4; RFC5; RPA1; RPA2; RPA3; LOC652672; LOC652857; GTF2H2B; TCEA1; XPA; XPC; CCNH
NHEJ(hsa03450_KEGG_Non_Homologous_End_Joining)	RAD50; DNTT; FEN1; XRCC6; POLL; POLM; LIG4; MRE11A; PRKDC; DCLRE1C; LOC731751; XRCC4; XRCC5; NHEJ1
SSB(GO:0003697_Single_Stranded_DNA_Binding)	ERCC1; ERCC4; ERCC5; FUBP1; HMGB2; HNRNPA1; HNRNPA2B1; HNRPDL; IGHMBP2; MLH1; MSH2; MSH3; MYT2; PCBP1; PMS2; POT1; PURA; PURB; RAD23A; RAD23B; RAD51; RAD51AP1; RBMS1; RPA1; RPA2; RPA3; RPA4; SUB1; TERF2; TERF2IP; TP53; TREX1; WBP11; XPC; YBX1

DDR(merged)	CCNO; POLD3; FEN1; SMUG1; APEX1; LIG1; LIG3; MPG; MUTYH; NTHL1; OGG1; PCNA; POLB; POLD1; POLD2; POLD4; TDG; XRCC1; MBD4; TCEA1; ERCC8; LOC652857; RPA3; RPA3; DDB2; POLR2E; POLR2H; RFC2; XPA; RFC3; ERCC1; RPA2; RPA2; CCNH; XAB2; ERCC6; POLE; ERCC2; RAD23B; POLR2J; POLR2G; DDB1; ERCC5; RPA1; RPA1; LOC652672; GTF2H2; POLR2B; ERCC4; POLR2L; POLR2A; RFC4; GTF2H1; ERCC3; RFC5; GTF2H4; GTF2H2B; POLR2D; POLR2I; CDK7; GTF2H3; POLE2; XPC; POLR2C; MNAT1; POLR2K; POLR2F; MSH3; MSH2; SSBP1; RFC1; EXO1; MLH1; RPA4; PMS2; MSH6; MLH3; PCBP1; PURB; ERCC1; HMGB2; IGHMBP2; RBMS1; RAD23B; HNRNPA2B1; TP53; ERCC5; TERF2IP; PURA; TREX1; ERCC4; WBP11; POT1; RAD51AP1; MYT2; RAD23A; HNRPDL; XPC; FUBP1; RAD51; RAD51; SUB1; HNRNPA1; YBX1; TERF2; RAD50; RAD50; H2AFX; LIG4; PRKDC; XRCC4; LOC651610; NBN; NBN; XRCC6; BRIP1; RAD52; RAD52; TP53BP1; BRCA1; MRE11A; MRE11A; MDC1; TDP1; LIG1; BRCA2; BRCA2; LOC389901; ATM; XRCC5; RAD54L; SHFM1; POLD3; TOP3A; XRCC3; POLD4; RAD51B; SSBP1; TOP3B; RAD51D; MUS81; POLD1; EME1; RPA4; RAD54B; RAD51C; XRCC2; BLM; POLD2; LIG4; PRKDC; NHEJ1; XRCC4; FEN1; POLM; XRCC6; DNTT; LOC731751; POLL; DCLRE1C; XRCC5; FANCA; FANCC; FANCD2; FANCE; FANCB; FANCF; FANCG; ZBTB32; UBE2T; ATM; ATR; FANCL; FANCM; RPS27A; LOC648152; LOC651610; LOC651921; BRCA1; BRCA2; RPS27AP11; UBA52; RPS27AP11; USP1; PALB2; C17orf70; C19orf40
